# Morphological Variations of the Mandibular Lingula: Clinical Implications for Surgical and Anesthetic Strategies in Adult Patients

**DOI:** 10.7759/cureus.79632

**Published:** 2025-02-25

**Authors:** Yasir H Elhassan

**Affiliations:** 1 Department of Basic Medical Sciences, Taibah University, Madinah, SAU

**Keywords:** anatomical variations, anesthetic techniques, clinical anatomy, inferior alveolar nerve, mandibular foramen, surgical procedures

## Abstract

Background: The mandibular lingula is an important anatomical landmark. It is also of dental, surgical, and radiological importance due to the existence of the inferior alveolar nerve and the mandibular foramen. Therefore, studies about morphological variations significantly improve the quality of surgical and anesthetic interventions.

Objective: This study explores the lingula's morphological variations in adult human mandibles and their importance in surgical and anesthetic procedures.

Materials and methods: A descriptive anatomical study was performed using 100 dry adult human mandibles (200 sides) obtained from three different Saudi university museums; all mandibles are of Indian origin.

The sample consisted of 80 male and 20 female mandibles. Using visual assessment and statistical evaluation, lingulae were classified into four types: triangular, truncated, nodular, and assimilated.

Results: Among the 200 mandibular sides examined, the triangular lingula was identified in 108 (54%) sides, with a variant exhibiting a narrow, flat, and more pointed apex observed unilaterally in 10 (5%) sides. The truncated, nodular, and assimilated types were present in 44 (22%), 26 (13%), and 12 (6%) sides, respectively. A male predisposition was noted for bilateral occurrences of the triangular subtype, whereas a statistically significant higher incidence of the nodular type was observed among females. Furthermore, the lingula tips were predominantly directed posterosuperiorly toward the condyle, being observed in 183 (91.5%) of the 200 sides.

Conclusion: In conclusion, we note the uniqueness of the lingula variations found, emphasizing the importance of these considerations to practitioners during surgical procedures. A better understanding of anatomy will be essential when performing surgery and providing anesthesia.

Future studies should involve sophisticated imaging methods to better explain lingula morphology.

## Introduction

The mandibular lingula is a bony projection resembling a tongue located along the inner margin of the mandible's ramus [1]. The lingula is particularly important in several dental and surgical interventions because of its proximity to the lower alveolar nerve and the mandibular foramen [2].

The classification of the lingula in human mandibles into triangular, truncated, nodular, and assimilated types is based on distinct morphological features that have clinical significance in dental and surgical practices [1,2]. Each type exhibits unique characteristics that can aid in anatomical identification and surgical planning.

The triangular lingula has the shape of a triangle with a broad base that narrows into a pointed apex. This type is the most frequently seen by populations worldwide, approximately 68.5%. In addition, the triangular shape is an important anatomical feature assisting dental treatment [1,3]. It is usually found just above the mandibular foramen and helps locate the area for blocking anesthesia of the inferior alveolar nerve [3]. 

In contrast to the triangular type, the truncated lingula is a rounded rectangle or quadrilateral with a more squarish appearance [1]. This type is not very common, approximately 15.8% of the population, compared to the triangular type, and can differ in height and width [3]. Such forms of truncation may limit access to the mandibular foramen, which is important for local anesthetic injections and surgical access to the mandible [1]. 

The nodular lingula's most marked feature is its rounded, bulbous structure, which can vary greatly in size. This type is often expressed as a prominent nodule on the ramus of the mandible, and its irregularity can make surgical intervention difficult. A nodular lingula may be present alongside other features, suggesting anatomical variation in surrounding structures, which needs to be addressed in the surgical design [4]. 

Assimilated lingula fails to exhibit any features other than those of the ramus of the mandible. This is the least frequent type (4.8% of the population); its outline is smooth and ill-defined [3,5]. The integrated form can mask the lingula's identification during surgery and might require special imaging procedures for precise delineation [5].

The understanding of these morphological variations is essential for the clinicians dealing with its surgery, especially orodental surgeons, as it would determine the effectiveness of anesthesia and the outcomes of surgeries. Conclusively, it can be said that the lingula depends mostly on the location of the mandibular foramen and hence serves as a major point for many of such procedures as pain relief using local anesthesia and surgical treatment of neuralgias by performing nerve section surgery [1,6]. It is important to protect these structures from such injurious environments while performing osteotomies of the mandibles [7]. This study concerns the incidence and shape of the lingula in adult human mandibles, and such information is of clinical significance.

It is particularly evident in surgical techniques, such as the sagittal ramus split, wherein one of the complications is that unwelcome fractures of the proximal segment may be seen, the incidence varying, according to authors, from 3% to 6.6% [8,9]. Similar anatomical conditions, such as the presence of a thin mandibular ramus along with a high-positioned lingula on the ramus, may predispose all fractures to such tendencies. This mandible configuration compresses the available cancellous bone and increases the fracture risk. Therefore, a modification of the sagittal split is preferred in situations of high lingulae or thin rami to reduce these effects [2,10].

The deep mandibular ramus is devoid of inconspicuous fat and contains the mandibular foramen, which is oriented centrally within the enlarged buccal component and communicates with the curved course of the mandible, which in turn contains neurovascular ampules of inferior alveolar nerve and vessels. These extensions arise from the outer periodontal membrane that surrounds the root and the bony sockets that house the teeth and other related structures to sustain the teeth functions [11,12]. Then, beyond the second premolar tooth, the canal divides into the mental and incisive canals, with the mental canal finishing at the mental foramen and the incisive canal running beneath the incisor teeth [13]. The lingula traverses the front edge of the mandibular foramen, while the sphenomandibular ligaments are attached to the posterior part of the bony structures. These ligaments are also in close proximity to the mylohyoid nerve and veins that pass via the mylohyoid muscle [14]. In addition, the gender-based variance in the everted angle of the female mandible calls for accurate comprehension of the anatomical orientations in procedures such as inferior alveolar nerve block and osteotomies since they require considerable accuracy in surgery and foresight with regard to the surgery [15].

The sphenomandibular ligament, a flexible, thin tissue band located near the sphenoid bone’s spinal process (and distinct from the temporomandibular joint) [16,17], exhibits an angular course that interacts with the lingula of the mandibular foramen before flattening. Moreover, the ligament is perforated by the mylohyoid nerve and vessels coursing through a narrow groove that extends anteriorly from the mandibular foramen beneath the mylohyoid line [18,19]. Understanding the sphenomandibular ligament’s role and its relationship with the lingula and adjacent anatomy is crucial for comprehensive surgical planning, as these features can influence both anesthetic efficacy and procedural outcomes [19,20].

This study attempts to describe the morpho-functional types of the lingulae and their significance, providing surgeons with reliable anatomical coordinates for planning complex mandibular interventions. Evaluating these anatomical variations preoperatively can enhance surgical outcomes and reduce complications, emphasizing the importance of a thorough patient-specific anatomical assessment immediately before the procedure.

## Materials and methods

Study design and setting

This descriptive anatomical study was performed on 100 dry adult human mandibles, providing a total of 200 mandibular sides, obtained from the Faculty of Medicine Museums at Taibah University (Al Madinah Al Munawarah), Umm Al-Qura University (Makkah-Al Mokarramah), and King Abdul Aziz University (Jeddah), Saudi Arabia. All specimens were sourced from a single supplier in India to ensure demographic and geographic consistency; additional details, such as collection dates and environmental conditions, can be provided if available.

Sample size and selection criteria

A total of 100 mandibles (80 male and 20 female) were analyzed. The study included intact dry adult human mandibles with clearly identifiable anatomical landmarks essential for assessing lingula morphology. Mandibles exhibiting fractures, significant wear, pathological deformations, or missing/damaged regions that could obscure key anatomical features were excluded (five mandibles were excluded out of 105).

Sex determination and validation

In order to ensure precise categorization of the mandibles by sex, we employed a multi-modal approach that combined both archival data and direct osteometric evaluation. Initially, the documented sex provided by the respective Faculty of Medicine Museums served as baseline demographic information. This preliminary identification was subsequently validated by examining key morphological features - such as the robustness of the mandibular ramus, gonial angle, and chin morphology - under standardized conditions using calibrated digital calipers, with measurements compared against benchmarks reported by Thakre et al. [1] and Tuli et al. [3]. To enhance reliability, two independent observers reviewed the archival records and conducted confirmatory assessments of the morphological markers; any discrepancies between the historical records and the observed osteometric parameters were resolved through consensus discussions. This rigorous cross-verification process, consistent with techniques outlined by Fernandes et al. [2], ensured that our gender-specific analyses of lingula morphology were founded on a robust and validated categorization system.

Data collection and morphological analysis

Mandibles were examined under standardized lighting and magnification conditions, and high-resolution digital photographs were taken using a Canon (Tokyo, Japan) EOS 5D Mark IV camera equipped with a Canon EF 100mm Macro lens with standardized settings to document morphological details. Two independent observers evaluated each specimen and classified the lingula into one of four morphological types: Triangular (with two subtypes: one with a narrow, rounded/pointed apex and another with a narrow, flat, more pointed apex), Truncated, Nodular, and Assimilated [1,2]. Discrepancies between observers were resolved through consensus discussions.

Statistical analysis

Data were entered and processed using SPSS Statistics Version 28 (IBM Corp., Armonk, NY, USA). Descriptive statistics were calculated, and the distribution of each lingula type was expressed in the “n (%)” format. Gender differences, as well as bilateral versus unilateral occurrences, were analyzed using chi-square tests or Fisher's exact tests (when cell counts were low), with a p-value of <0.05 considered statistically significant. In addition, cross-tabulation analyses were conducted to assess associations between lingula morphology subtypes and the directional orientations of their tips relative to the mandibular condyle.

Quality assurance

To ensure data accuracy and consistency, all specimens were independently evaluated by two experienced observers following a calibration session intended to harmonize the classification criteria. Data were then double-entered and cross-verified by a third reviewer to resolve any discrepancies. All photographic documentation was meticulously reviewed for clarity and accuracy.

Ethical considerations and IRB approval

Although this study used archival skeletal specimens, ethical clearance was obtained. The study was reviewed and approved by the Institutional Review Board (IRB) of Taibah University College of Medicine under the approval number TU-23-01, dated 10/4/2023, in compliance with all institutional ethical standards.

## Results

Lingual morphology

**Figure 1:**
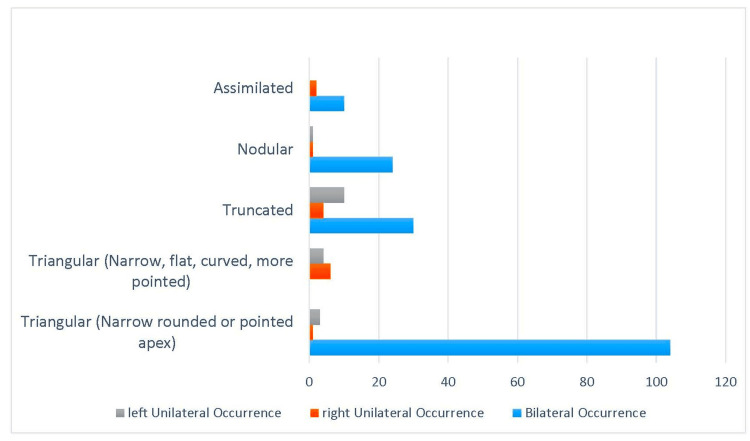
Distribution of Lingula Types by Side This composite illustration presents the morphological distribution of the mandibular lingula among a total of 200 sides. The x-axis displays the various lingula types—namely, Triangular (narrow, rounded, or pointed apex), Triangular variant (narrow, flat, curved, more pointed), Truncated, Nodular, and assimilated—while the y-axis indicates the number of occurrences. Specifically, the triangular lingula was observed on 108 sides, the triangular variant on 10 sides, the truncated type on 44 sides, the nodular type on 26 sides, and the assimilated type on 12 sides. Notably, most lingulae, especially the triangular type, exhibited a postero-superior orientation relative to adjacent anatomical landmarks, emphasizing their clinical relevance.

**Figure 2:**
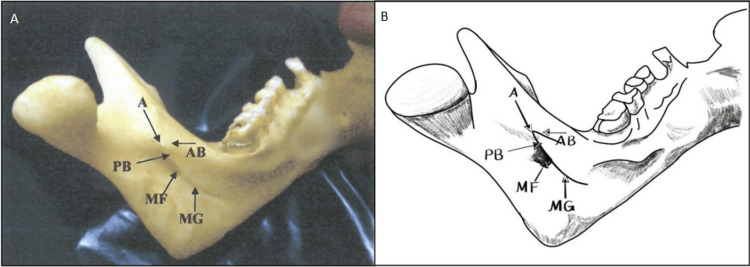
Triangular Lingula Panels A and B illustrate the typical triangular lingula. In Panel A, a high-resolution photographic image displays the triangular lingula with a broad base and a narrow, pointed apex. Panel B provides a complementary diagrammatic illustration that labels critical anatomical landmarks such as A: Apex, PB: Posterior Border, AB: Anterior Border, MF: Mandibular Foramen, and MG: Mandibular groove. Together, these figures underscore the clinical importance of the triangular form for precise localization during inferior alveolar nerve blocks and osteotomy procedures. Panel B credits: Dr. Liena Babiker Mekki, created using Procreate; permission for use granted.

The study documented four distinct types of lingulae in adult human mandibles. The first type, the triangular lingula, presents with a wide base and a narrow, rounded, or pointed apex and was observed in 108 (54%) of the 200 sides examined. Of these, 104 sides were bilateral (from 52 mandibles), while four sides exhibited a unilateral occurrence (with one on the right and three on the left), and when unilateral, the contralateral side showed a truncated lingula (Table 1, Figures 1, 2).

**Table 1:** Distribution of Lingula Types by Side This table represents the distribution of different lingula types based on their occurrence in adult human mandibles. Out of 200 mandibular sides analyzed, 84% exhibited bilateral occurrence, while 16% were unilateral. The triangular lingula with a narrow, rounded, or pointed apex was the most predominant, identified in 54% of sides, with 96.3% of these cases showing bilateral symmetry. In contrast, the triangular variant with a narrow, flat, curved, and more pointed apex was observed exclusively unilaterally in 5% of the sides. Additionally, the truncated type was present in 22% of cases (approximately 68.2% bilateral and 31.8% unilateral), the nodular type in 13% (92.3% bilateral, 7.7% unilateral), and the assimilated type in 6% (83.3% bilateral, 16.7% unilateral).

Lingula Type	Bilateral Occurrence	Unilateral Occurrence	Total Sides (%)
Triangular (Narrow rounded or pointed apex)	104 sides	Right: 1, Left: 3	108 (54%)
Triangular (Narrow, flat, curved, more pointed)	0	Right: 6, Left: 4	10 (5%)
Truncated	30 sides	Right: 4, Left: 10	44 (22%)
Nodular	24 sides	Right: 1, Left: 1	26 (13%)
Assimilated	10 sides	Right: 2, Left: 0	12 (6%)
Total	168	32	1

**Figure 3:**
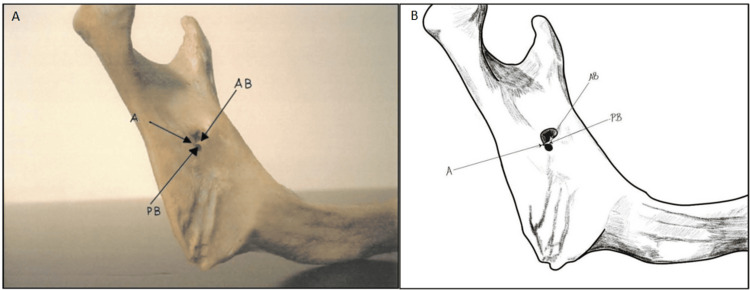
Variant of the Triangular Lingula Panels A and B demonstrate a less common variant of the triangular lingula characterized by a narrow, flat, and more pointed apex. The photographic image (Panel A) and the accompanying diagram (Panel B) elaborate on the free anterior and posterior borders visible in this variant. The subtle differences highlighted in these images explain how this variant may affect the accuracy of landmark-based surgical approaches compared to the classical triangular form. A: Apex, AB: Anterior border, PB: Posterior border. Panel B credits: Dr. Liena Babiker Mekki, created using Procreate; permission for use granted.

In addition, a variation of the triangular form, characterized by a narrow, flat, and more pointed apex, was noted unilaterally on 10 (5%) sides (six on the right and four on the left) (Table 1, Figures 1, 3).

**Figure 4:**
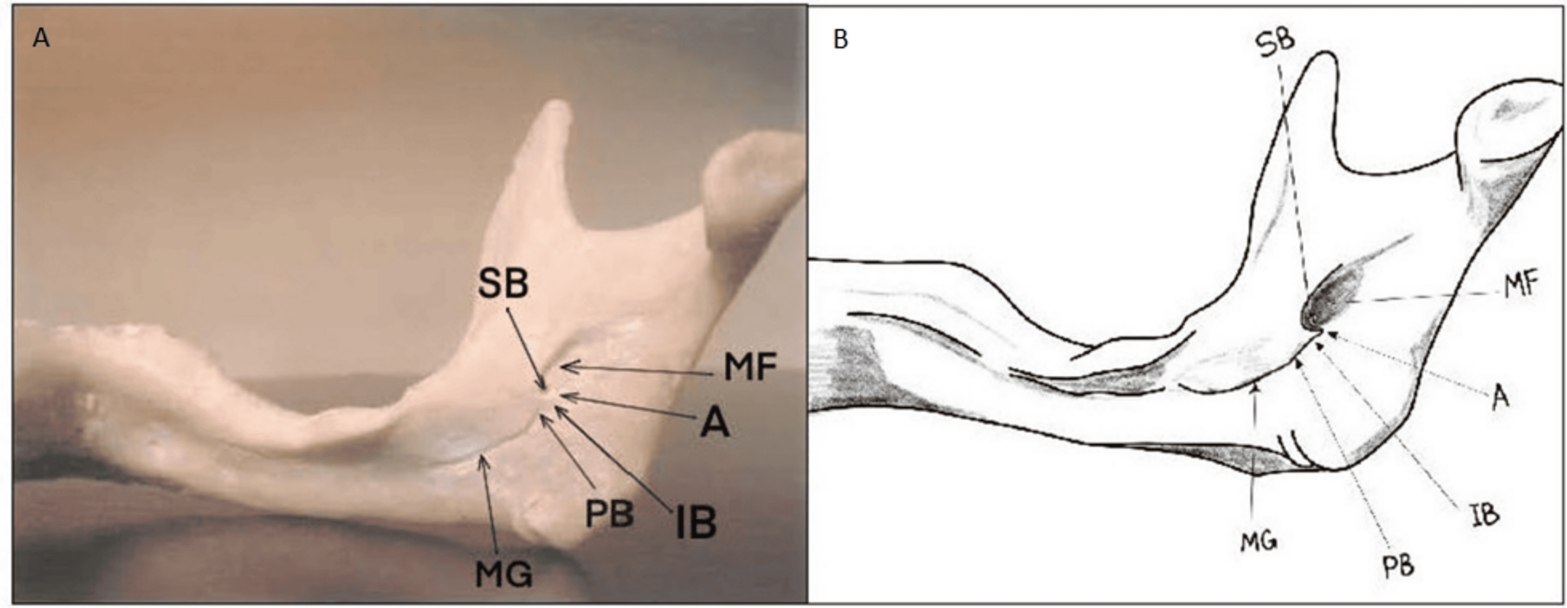
Truncated Lingula Panels A and B focus on the truncated lingula. The images display its quadrangular appearance with a relatively squared-off upper contour. The combination of the clinical photograph (Panel A) and the diagrammatic illustration (Panel B) provides insight into how the altered shape might affect the spatial relationship with the mandibular foramen—critical for planning local anesthetic injections and surgical interventions. A: Apex, PB: Posterior Border, AB: Anterior Border, MF: Mandibular Foramen, MG: Mandibular groove. Panel B credits: Dr. Liena Babiker Mekki, created using Procreate; permission for use granted.

The second type, the truncated lingula, was identified on 44 (22%) sides and is distinguished by a quadrangular appearance; it occurred bilaterally on 30 sides and unilaterally on 14 sides (four on the right and 10 on the left) (Table 1, Figures 1, 4).

**Figure 5:**
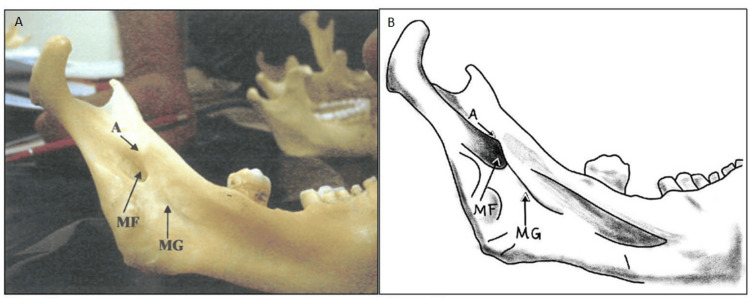
Nodular Lingula Panel A is a photographic image of the nodular lingula, illustrating its variable, rounded shape and near fusion to the mandibular ramus, except for the distinct apex. Panel B is a diagrammatic illustration of the nodular lingula, emphasizing its bulbous appearance and integration with the surrounding bone structure. A: Apex, MF: Mandibular Foramen, MG: Mandibular Groove. The nodular lingula is distinguished by its rounded, bulbous morphology and its frequent near fusion with the ramus. This variation can complicate the identification of the mandibular foramen. Panel B credits: Dr. Liena Babiker Mekki, created using Procreate; permission for use granted.

The third type, the nodular lingula, which exhibits variable size and is nearly fused to the ramus except at its apex, appeared on 26 (13%) sides, with a bilateral presentation on 24 sides and unilateral occurrence on two sides (one right, one left) (Table 1, Figures 1, 5).

**Figure 6:**
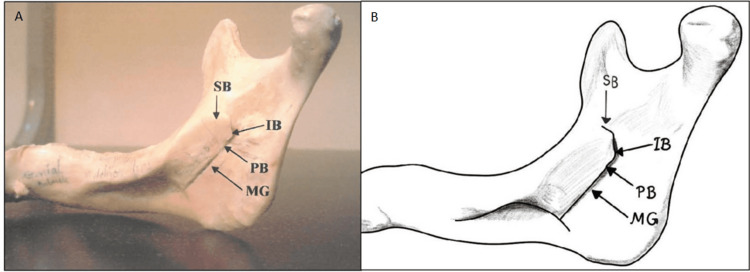
Assimilated Lingula Panel A is a photographic image of the assimilated lingula showing its seamless integration within the mandibular ramus. Panel B is a diagrammatic illustration highlighting the assimilated lingula’s smooth contour and lack of a distinct tip. SB: superior Border, IB: inferior Border, PB: Posterior border, MF: Mandibular Foramen, MG: Mandibular groove. The assimilated lingula is characterized by its complete incorporation into the mandibular ramus, blending seamlessly with the surrounding bone. This unique morphology may complicate the identification of the mandibular foramen during clinical procedures. Panel B credits: Dr. Liena Babiker Mekki, created using Procreate; permission for use granted.

Lastly, the assimilated lingula, completely incorporated within the ramus, was found on 12 (6%) sides, occurring bilaterally on 10 sides and unilaterally on two sides (both on the right) (Table 1, Figures 1, 6).

Gender variation

**Figure 7:**
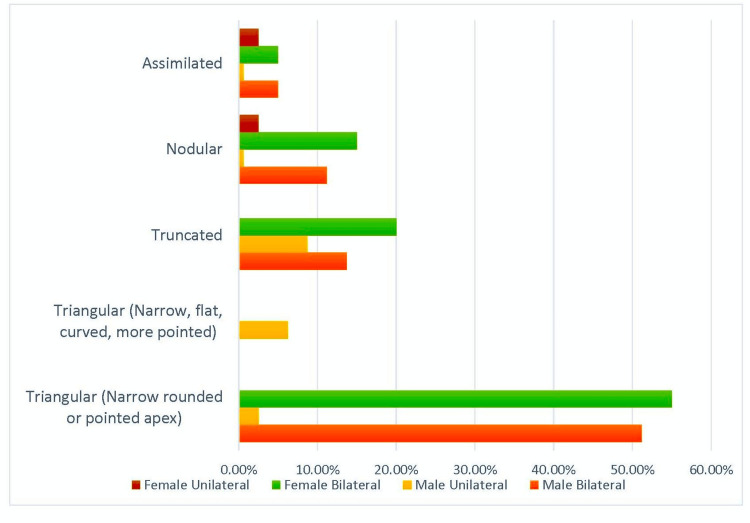
Gender-Specific Distribution of Mandibular Lingula Types This figure illustrates the gender-specific distribution of mandibular lingula types observed on 200 sides. The x-axis represents the different lingula categories—namely, the triangular type (with a narrow, rounded, or pointed apex), the triangular variant type (with a narrow, flat, curved, and more pointed apex), truncated, nodular, and assimilated lingulae—while the y-axis indicates the frequency (expressed as percentages) of occurrences.

The distribution of lingula types showed notable gender-based differences. In males, the triangular type with a narrow, rounded, or pointed apex was predominant, occurring bilaterally in 51.2% and unilaterally in 2.5% of cases. A variation of this type, featuring a narrow, flat, and more pointed apex, was observed unilaterally in 6.2% of males. Truncated lingulae were found bilaterally in 13.7% and unilaterally in 8.7% of males. Nodular lingulae occurred bilaterally in 11.2% and unilaterally in 0.6% of males, while assimilated lingulae were evident bilaterally in 5% and unilaterally in 0.6% of males (Table 2, Figure 7).

**Table 2:** Gender-Specific Distribution of Mandibular Lingula Types This table displays the distribution of lingula morphological types according to gender among 200 mandibular sides. The columns "Male Bilateral" and "Male Unilateral" indicate the frequency and percentage of each lingula type observed bilaterally or unilaterally among male specimens, while "Female Bilateral" and "Female Unilateral" report the corresponding data for female specimens. For example, the triangular lingula with a narrow, rounded, or pointed apex is the most common type, occurring in 82 bilateral and four unilateral instances in males (totaling 108 sides or 54% of all sides), and exclusively bilaterally in females (22 sides, 55% of female cases). Similarly, other lingula variations, such as the truncated, nodular, and assimilated types, are detailed by their occurrence pattern and respective percentages. This descriptive breakdown highlights potential gender-related anatomical differences in lingula morphology, providing insights into both bilateral symmetry and unilateral occurrence across the study sample.

Lingula Type	Male Bilateral	Male Unilateral	Female Bilateral	Female Unilateral	Total Sides (%)
Triangular (Narrow rounded or pointed apex)	82 (51.2%)	4 (2.5%)	22 (55%)	0	108 (54%)
Triangular (Narrow, flat, curved, more pointed)	0	10 (6.2%)	0	0	10 (5%)
Truncated	22 (13.7%)	14 (8.7%)	8 (20%)	0	44 (22%)
Nodular	18 (11.2%)	1 (0.6%)	6 (15%)	1 (2.5%)	26 (13%)
Assimilated	8 (5%)	1 (0.6%)	2 (5%)	1 (2.5%)	12 (6%)

Among females, the triangular (narrow, rounded, or pointed apex) type was bilateral in 55% of cases, while nodular lingulae showed a higher incidence, with bilateral occurrence in 15% and unilateral in 2.5%. Truncated and assimilated types were also documented but less frequently (Table 2, Figure 7).

Direction and attachment of lingula tips

The study revealed significant differences in the anatomical orientation of the lingula tips across the various observed types. For the triangular lingula (Type 1), which was present on 118 (59%) of the 200 sides, two distinct subtypes were identified based on tip direction. The predominant subtype, Type 1a, exhibited tips directed postero-superiorly toward the condyle and was observed in 108 (91.5%) of the 118 Type 1 sides (Table 3, Figure 2).

**Table 3:** Distribution of Lingula Types and their Subtypes Based on the Direction and Attachment of their Tips This table summarizes the distribution of mandibular lingula subtypes based on tip orientation and attachment across 200 sides. Type 1 (Triangular) lingulae were identified in 118 sides (59% of cases) and are subdivided into Type 1a (108 sides, representing 91.5% of Type 1 and 54% of total sides) with a predominant postero-superior orientation, and Type 1b (10 sides, 8.5% of Type 1 and 5% of total sides) with an alternative orientation. Type 2 (Truncated) lingulae were observed in 44 sides (22% of cases) and include Type 2a (32 sides, 72.7% of Type 2 or 16% overall) with a slightly convex superior border, Type 2b (8 sides, 18.2% of Type 2 or 4% overall) with a straight border, and Type 2c (4 sides, 9.1% of Type 2 or 2% overall) with a concave border.

Lingula Type	Subtype	Number of Sides	Percentage of Subtype	Percentage of Total Sides
Type 1 (Triangular)	Type 1a	108	91.5% of Type 1	54.00%
	Type 1b	10	8.5% of Type 1	5.00%
Total for Type 1	-	118	-	59.00%
Type 2 (Truncated)	Type 2a	32	72.7% of Type 2	16.00%
	Type 2b	8	18.2% of Type 2	4.00%
	Type 2c	4	9.1% of Type 2	2.00%
Total for Type 2	-	44	-	22.00%

In contrast, the less common Type 1b variant, with the tip oriented towards the posterior border, was noted in only 10 (8.5%) of the Type 1 cases (Table 3, Figure 3). Regarding the truncated lingula (Type 2), which was identified on 44 (22%) of the sides, the tip is positioned between the superior and inferior borders, forming part of a structure defined by its three borders (superior, inferior, and posterior). Within this type, 32 (72.7%) of the 44 cases were classified as Type 2a, featuring a slightly convex superior border (Table 3, Figure 4); eight (18.2%) were Type 2b, with a straight superior border, and four (9.1%) were Type 2c, where the superior border was concave. In contrast, the nodular and assimilated lingula types did not display a distinct tip due to their integration into the mandibular ramus and varying degrees of fusion with the surrounding bone.

These findings highlight the diverse morphological orientations of lingulae, underscoring the importance of understanding these variations for enhancing the precision of surgical and anesthetic procedures involving the mandible.

## Discussion

The differing forms of the mandibular lingula are of considerable importance for surgical and anesthetic procedures. For instance, in orthognathic surgery, the location and form of the lingula can significantly affect the surgical approach and the risk of nerve injury. Similarly, in local anesthesia administration, the lingula's form can influence the success and safety of the procedure [21,22]. This study noted four forms: triangular, truncated, nodular, and assimilated, with the triangular form being the most common at 54%. This finding is consistent with earlier studies [4,23]. A thorough study by Tuli et al. [3] of 165 dry adult mandibles found that the triangle form had approximately 68.5% of cases compared to 15.8% for truncated, 10.9% for nodular, and 4.8% for assimilated forms. In the same spirit, Thakre et al. [1] noted the prominence of triangular lingulae (110 specimens) and lower incidence of other types, while Asdullah et al. [4] further confirmed that the triangular and truncated shapes form the most commonly encountered configurations in human mandibles. These studies collectively establish that the classification of the lingula based mainly on its shape is accurate and has broad applicability in different populations; the slight dissimilarity in the percentages could be due to ethnic group diversity.

This classification carries much more than academic importance; it has clinical significance. The lingula forms a considerable morphologic landmark for the inferior alveolar nerve block (IANB) and surgical procedures such as sagittal split ramus osteotomy (SSRO). Its variations possess the ability to increase the likelihood of certain morbidities like nerve injury, hemorrhage, and adverse fractures [10,24]. For instance, a high-positioned or irregularly shaped lingula, especially in patients with slender mandibular ramus, could lead to changes in surgical technique to prevent fractures [23,25]. Furthermore, the anterior border of the lingula was found to be predominantly oriented posterosuperiorly toward the condyle (Type 1a), with a notable 91.5% of the assessed population possessing this feature, which is bone fide; the reason being the orientation affects the direction of the surgical instruments and the position of the mandibular foramen which is critical for effective IANB [21,22,24].

The identification of the Type 1b variant form characterized by a posteriorly oriented tip- is particularly relevant. In instances where this configuration is present, surgeons may need to adjust their surgical techniques to minimize the risk of damaging the inferior alveolar nerve, especially during procedures like sagittal ramus split osteotomy. Similarly, during local anesthesia administration, the lingula's form, including the Type 1b variant, may influence both the precision and safety of the procedure [16].

Variations in lingula morphology due to gender further stress the necessity for specialized surgical techniques. In our investigation, it was noted that females had a higher prevalence of nodular type, suggesting that anatomical factors related to sex should be included in surgical and anesthetic strategies [4,25]. This leads to a difference in the accuracy of nerve blocks [26,27] and increases the value of preoperative evaluation and appropriate technique modification.

Some other studies have covered the shape of the lingula, but it seems to be incorporated into the existing classification system much more efficiently than others [1,3-6].

Although the assimilated and nodular forms may not be the most common, they are clinically relevant because their typical patterns could mask familiar bony landmarks and hinder surgical access, thereby increasing the complication risks during mandibular operations [5,6]. Mastery of these differences improves the reliability of the surgical outcomes and the effectiveness of the anesthetic methods employed.

Limitations

The use of only dry adult mandibles may not fully reflect the existing functional anatomical relationships of more living subjects, which could affect the application of these results to real-world settings. Furthermore, this sample was made up entirely of Indian mandible samples from three Saudi university museums, which may not represent other populations. Moreover, the small sample size may not depict the complete range of morphological diversity, and further studies are needed with larger, more diverse samples, as well as confirmatory imaging using cone beam computed tomography (CBCT) to address these issues.

## Conclusions

Winding up, the morphological changes of the mandibular lingula are very critical for improving surgical and anesthetic practices. The predominance of the triangular type, especially with marked differences between the genders, calls for treatment strategies that consider these anatomical factors. A solid understanding of these morphological variations will help not only in orienting to the landmarks but also in estimating the surgical risks in IANB and SSRO. It is crucial that further investigations are conducted with more sophisticated imaging techniques and broader ethnic scopes in order to fully delineate these variations and improve clinical guidelines in terms of safety and effectiveness within dentistry and maxillofacial surgery.
